# The Effects of Sleep Quality on Dream and Waking Emotions

**DOI:** 10.3390/ijerph18020431

**Published:** 2021-01-07

**Authors:** Francesca Conte, Nicola Cellini, Oreste De Rosa, Marissa Lynn Rescott, Serena Malloggi, Fiorenza Giganti, Gianluca Ficca

**Affiliations:** 1 Department of Psychology, University of Campania L. Vanvitelli, Viale Ellittico 31, 81100 Caserta, Italy; oreste.derosa@unicampania.it (O.D.R.); marissalynn.rescott@unicampania.it (M.L.R.); gianluca.ficca@unicampania.it (G.F.); 2 Department of General Psychology, University of Padova, Via Venezia 8, 35131 Padova, Italy; cellini.nicola@gmail.com; 3 Department of Biomedical Sciences, University of Padova, Via Ugo Bassi 58/B, 35131 Padova, Italy; 4 Padova Neuroscience Center, University of Padova, Via Giuseppe Orus 2, 35131 Padova, Italy; 5 Human Inspired Technology Center, University of Padova, Via Luzzatti 4, 35121 Padova, Italy; 6 Department NEUROFARBA, University of Firenze, Via di San Salvi 12, 50135 Firenze, Italy; serena.malloggi@unifi.it (S.M.); fiorenza.giganti@unifi.it (F.G.)

**Keywords:** dreaming, emotions, sleep quality, good sleepers, poor sleepers

## Abstract

Despite the increasing interest in sleep and dream-related processes of emotion regulation, their reflection into waking and dream emotional experience remains unclear. We have previously described a discontinuity between wakefulness and dreaming, with a prevalence of positive emotions in wakefulness and negative emotions during sleep. Here we aim to investigate whether this profile may be affected by poor sleep quality. Twenty-three ‘Good Sleepers’ (GS) and 27 ‘Poor Sleepers’ (PS), identified through the Pittsburgh Sleep Quality Index (PSQI) cut-off score, completed three forms of the modified Differential Emotions Scale, assessing, respectively, the frequency of 22 emotions over the past 2 weeks, their intensity during dreaming and during the previous day. The ANOVA revealed a different pattern of emotionality between groups: GS showed high positive emotionality in wakefulness (both past 2 weeks and 24 h) with a significant shift to negative emotionality in dreams, while PS showed evenly distributed emotional valence across all three conditions. No significant regression model emerged between waking and dream affect. In the frame of recent hypotheses on the role of dreaming in emotion regulation, our findings suggest that the different day/night expression of emotions between groups depends on a relative impairment of sleep-related processes of affect regulation in poor sleepers. Moreover, these results highlight the importance of including sleep quality assessments in future dream studies.

## 1. Introduction

The interaction between sleep and affective brain function has received attention only in the last couple of decades. As pointed out by Walker and van der Helm [1], this delay appears surprising in light of two observations. On one hand, there is significant overlap between sleep physiology and the brain networks and neurochemical processes involved in affective modulation; in addition, sleep dysfunctions co-occur with remarkable frequency in most affective psychiatric disorders [1].

Despite the dearth of past research on the topic, recent work has begun to point out the importance of sleep for the regulation of emotions (see, e.g., [2] for a recent review). The role of sleep in affective processing is generally explained in light of the peculiar neurophysiology of sleep, and REM sleep in particular (see, e.g., [1,3]). In fact, this sleep state is associated with a relative deactivation of several areas of the neocortex [4,5], paralleling an increased activity in subcortical regions [4,6]. This pattern of activation, accompanied by the distinctive neurochemical balance occurring during REM sleep [7,8], is believed to provide optimal conditions for offline processing of emotional information.

In line with the prominent involvement hypothesized for REM sleep in emotional processing, the most recent theoretical approaches propose an important role of mental activity occurring during sleep (i.e., dreaming, according to Schredl and Wittman’s definition [9]) in these complex regulatory processes. At the biological level, it is supported by the existence of largely overlapping neural networks sustaining both (REM) dreaming and emotional processing (extensively reviewed in [10]). Indeed, several models propose that dreaming actively participates in the regulation of prior daytime emotions by facilitating the resolution of emotional conflicts [11,12], enhancing fear-extinction processes [3], and depotentiating the affective tone initially associated with waking events [1]. Another set of hypotheses focuses instead on the role of dreaming in optimizing affective reactions to future waking events: dreaming would allow an offline simulation of threatening or social episodes and a rehearsal of the corresponding threat- or social coping skills (respectively the “threat simulation theory” [13] and the “social simulation theory” [14]). Ultimately, both types of models converge in suggesting that waking and dream emotions are closely connected and that emotional processing occurring in dreams promotes adaptive behavioral responses to the challenges of waking life.

However, a clear understanding of the relationship between waking and dream emotions and their expression in subjective daytime consciousness and sleep mentation is still lacking. A recent study by our group [15] has addressed this issue in a sample of healthy adults: emotions of the last recalled dream, as well as those of the previous day and previous two weeks, were collected (through the modified Differential Emotions Scale, mDES [16,17]) and compared. Our findings mainly highlighted a discontinuity between waking and dream affect, with positive emotionality prevailing during the past two weeks as well as the day before the dream and reduced in the dream, while negative emotionality of the dream was similar to that of the preceding two weeks but significantly increased relative to the previous day. This interesting pattern of results opened the way to several hypotheses, such as the possibility that positive and negative emotions experienced in wakefulness may undertake different but parallel sleep-related regulation pathways.

As also suggested in the discussion of those findings [15], another intriguing hypothesis is that the relationships between waking and dream emotions (plausibly reflecting affective regulation processes) may be modulated by sleep quality. In fact, in the last couple of decades, a vast amount of research has focused on the effects of sleep disruption on several aspects of affective processing.

One night of sleep deprivation is sufficient to increase subjective reports of stress, anxiety, and anger in response to low-stress situations [18] and to increase impulsivity toward negative stimuli [19]. Moreover, after one night of sleep deprivation, subjects evaluated neutral pictures more negatively than control participants [20,21], independently of negative mood [20]. Impairments of emotion recognition [22] and expression [23] have been observed as well after single-night sleep deprivation.

Other studies provide evidence of emotional dysregulation following sleep deprivation using neural and physiological measures of emotionality. Enhanced amygdala reactivity in response to emotionally negative pictures, paralleled by a reduction of functional connectivity with medial prefrontal regions (believed to exert top-down regulatory control on the amygdala), has been detected after one night of sleep deprivation [24] as well as after five nights of sleep restriction [25]. Also, sleep loss has been shown to amplify pupil diameter responses during passive viewing of negative emotional pictures [26] and to increase sympathetic dominance of the autonomic nervous system, indexed by changes in heart rate variability [27].

An impact of sleep loss on affective processing has also been described in more ecologically relevant paradigms, i.e., based on cumulative sleep restriction protocols or on samples with impaired sleep quality. For instance, negative emotional changes have been reported in both adults [28] and adolescents [29] after several days of sleep restriction. Furthermore, poor subjective sleep quality has been associated with higher negative [30,31] and lower positive emotionality [30,31,32] and with decreased ability in cognitive reappraisal [33]. Habitual self-reported sleep quality has also been found to moderate the relationship between threat-related amygdala reactivity, negative affect, and perceived stress [34]. Furthermore, Tempesta et al. [21] showed that poor sleepers (classified through the Pittsburgh Sleep Quality Index, PSQI [35]) evaluated neutral pictures more negatively than good sleepers.

In sum, this brief review of data provides strong support to the idea that sleep disruption impairs affective regulation. In light of the aforementioned hypotheses on dreams as a reflection of ongoing emotional processing, dream emotions of individuals with disturbed sleep may represent an interesting object of study. The very few studies addressing this issue show that dreams of insomniacs [36,37,38] and narcolecptic subjects [39] are more negatively toned than those of good sleepers; also, nightmare frequency appears to be more elevated in individuals with poor sleep quality [40,41,42,43]. However, focusing exclusively on dream emotions, these studies do not allow the authors to make hypotheses on the possible differences between good and poor sleepers in emotion regulatory processes, which are probably better expressed in the relationships between waking and dream emotions rather than in dream emotions alone.

Indeed, several hypotheses on the presentation of waking and dream emotions in good and poor sleepers may be put forward. For instance, the profile of differences between daytime and dream emotionality observed in our previous study [15] could emerge in poor sleepers as well, indicating the presence of a similar pathway of affective processing notwithstanding the possible dysfunctionality of emotion regulation processes in poor sleepers observed in previous literature (e.g., [21,33]). Alternatively, poor sleepers could display an inverse pattern of emotionality in wakefulness and dreaming relative to good sleepers, with negative tone predominant in wakefulness and a positive rebound in sleep. Also, at variance with good sleepers, poor sleepers could manifest a more evenly distributed emotional tone (similar in both states of consciousness), and so on. The possibilities are multiplied when considering the time span over which these mechanisms unfold: for instance, each dream may process emotions experienced the day before, a few days before (in analogy with literature on the “dream lag” and “day-residue” effect [44,45]), or during wider daytime spans (e.g., the last few weeks, the general “time period”), etc.

Therefore, here we conduct an exploratory study to investigate the relationships between waking emotions and those of the subsequent night’s dreams in a sample of good and poor sleepers identified through the PSQI [35]. Specific aims of our study are:

1. to compare, between good and poor sleepers, the prevalent emotional valence of the dream with that of the previous day and previous weeks;
2. to assess the possibility that waking emotionality predicts dream emotionality in good and poor sleepers;
3. to confirm findings from previous literature on dream emotional valence in good and poor sleepers using an instrument, the mDES [16,17], which addresses a repertoire of emotions broader than the ones commonly used in dream literature.

## 2. Materials and Methods

### 2.1. Participants and Procedure

**Figure 1:**
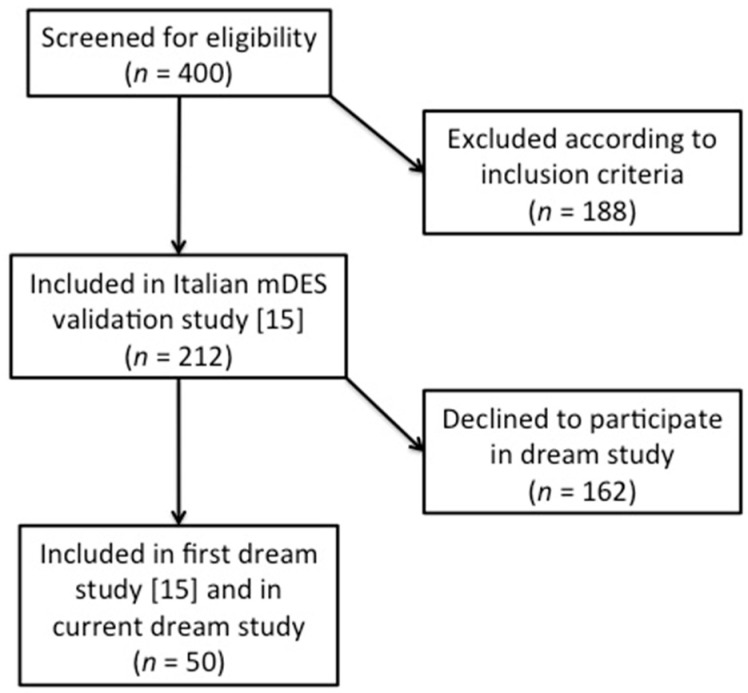
Flowchart of the participant recruitment and selection process.

Figure 1 displays the recruitment and selection process. Four hundred volunteers from the cities of Naples and Caserta (Italy) were screened through a brief ad-hoc interview to collect general demographic data and information on medical conditions and life habits. The interview was conducted via telephone by a psychologist from the Sleep Lab of the University of Campania. Two hundred and twelve healthy participants (163 F, 49 M; mean age: 25 ± 8 years) were thus selected for the study, according to the following inclusion criteria: age between 18 and 65 years; absence of any relevant somatic or psychiatric disorder; absence of any sleep apnea or respiratory disorder symptoms; having a regular sleep–wake pattern; absence of sleep disorders; no history of drug or alcohol abuse; limited caffeine (no more than 150 mg caffeine per day, corresponding to about three cups of espresso or one cup of American coffee) and alcohol (no more than 250 mL per day) consumption.

The whole selected sample (*N* = 212) participated in a larger study [15], which included a validation of the Italian version of the mDES [16,17]. Thus, two forms of the questionnaire (WAKE-24 h and WAKE-2 weeks, assessing the frequency of specific emotions over the past 2 weeks and their intensity in the past 24 h, respectively) were administered to participants along with the Mannheim Dream Questionnaire (MADRE [46]) to collect data on dream recall frequency and several variables related to dreams, and the PSQI [35], in its Italian version [47], to assess habitual subjective sleep quality.

Of the 212 participants included in the validation study, 50 (38 F, 12 M; mean age: 24.6 ± 6.4 years) volunteered to take part in a second phase of the study, i.e., the assessment of relationships between waking and dream emotions. Participants received 10 copies of the WAKE-24 h mDES, with the instruction to complete one each night at bedtime, referring to the emotions experienced during that particular day. This had to be done until the day they recalled a dream. On the morning they recalled a dream, they had to fill in the DREAM mDES, specifically referring to the emotions experienced during the dream. Data collection was thus ended as soon as the mDES ratings of one dream were provided by each participant.

While our previous study [15] focused on differences between waking and dream emotions in the general sample, this study analyses the same dataset with regard to sleep quality, i.e., by dividing the final sample (*N* = 50) into a group of ‘Good Sleepers’ and a group of ‘Poor Sleepers’ (GS and PS, respectively) based on the PSQI cut-off score (scores ≥ 5 indicate poor sleep quality [35]).

### 2.2. Instruments

1. Italian version of the mDES: The original mDES [16,17] consists of 20 items corresponding to 20 different emotions (10 positive and 10 negative) whose intensity over the past 24 h is rated on a five-point Likert scale (from 0 = Not at all, to 4 = Extremely). Each category is described by three adjectives (e.g., “Grateful, appreciative, or thankful”): for clarity purposes, throughout the manuscript the noun referring to the first of the three adjectives will be used to identify specific emotion categories (e.g., “Gratefulness”). The Italian version [15] includes two additional positive emotions (“sexual/desiring/flirtatious” and “sympathy/concern/compassion”), which were included in the earlier version of the instrument [16]. In addition to this standard version (labeled WAKE-24 h mDES [15]), two other forms of the scale were developed in our previous study [15], assessing, respectively, the frequency of each emotion over the past two weeks (WAKE-2 weeks mDES) and the intensity of emotions experienced during the last recalled dream (DREAM mDES). The specific instructions provided in the DREAM and the WAKE-24 h mDES versions are: “Please think back to how you have felt during your last recalled dream/last 24 h. Using the 0–4 scale below, indicate the greatest amount that you’ve experienced each of the following feelings.” As for the WAKE-2 weeks form, the instructions are: “Please think back to how you have felt during the past two weeks. Using the 0–4 scale below, indicate the frequency with which you’ve experienced each of the following feelings.” (from 0 = Never, to 4 = Very frequently). The mDES also allows the use of aggregate measures of positive and negative emotionality (the Positive Affect (PA) and Negative Affect (NA) subscales, i.e., average scores of the positive and negative emotion items, respectively), which have shown to have high internal reliability, ranging from 0.82 to 0.94 [48,49]. The scale has been validated on the Greek [50] and Italian [15] populations and has shown to have good psychometric properties in its various translations [15,50,51,52,53].
2. PSQI [35]: This questionnaire assesses sleep quality and disturbances over a 1-month time interval. It consists of 19 individual items which generate seven component scores: subjective sleep quality, sleep latency, sleep duration, habitual sleep efficiency, sleep disturbances, use of sleeping medication and daytime dysfunction. The sum of scores for these seven components yields one global score, ranging from 0 to 21, with 5 as a cut-off score which allows to differentiate good from poor sleepers [35] (higher scores indicate worse sleep quality). Here we use the Italian version of the PSQI [47], which has been validated on the Italian population [47].
3. MADRE questionnaire [46]: This questionnaire measures several variables related to dreams such as frequency of dream recall, nightmares and lucid dreaming, attitude towards dreams and the effects of dreams on waking life. We report frequency of dreams, lucid dreams, and nightmares, as well as intensity of the dream experience, attitude towards dreams and correlates of dreams (the sum of items 13-14-15-16-17), all referring to how the contents of dreams are used in terms of problem solving and creativity (see [54]).

### 2.3. Data Analysis

Differences between GS and PS in age, gender distribution and MADRE scores were assessed using independent *t*-test, χ^2^ (for categorical data) and Mann–Whitney test (for ordinal data). To assess the differences between groups in emotional valence of dreams and previous wakefulness, we conducted a 2 (Group: GS, PS) × 3 (Condition: WAKE-2 weeks, WAKE-24 h, DREAM) mixed ANOVA, with Δ mDES score (PA minus NA, i.e., an aggregate measure of valence, with positive values indicating positive valence and negative values indicating negative valence) as dependent variable. We used η^2^p as a measure of effect size and the Holm test for post-hoc analysis.

Also, in order to explore the potential predictors of dream emotions, we conducted, separately for GS and PS, a linear regression with DREAM Δ scores as dependent variables and WAKE-2 weeks and WAKE-24 h Δ scores as predictors. For each significant predictor, we reported the unstandardized (b) and the standardized (β) coefficient. All analyses were conducted using JAMOVI 1.2.27 and a *p* < 0.05 was considered statistically significant.

## 3. Results

### 3.1. Descriptives

The sample was made up of 38 females (76%) and 12 males (24%), with an age range of 19 to 52 years.

A total of 50 DREAM mDES and 50 WAKE-2 weeks mDES (one per participant) were collected. As for the WAKE-24 h version, 84 scales were collected in total (50 referring to the day immediately preceding the dream and the remaining referred to the previous days); in fact, 30 participants (60%) recalled a dream after 1 night, 6 participants (12%) after 2 nights, and the remaining 14 (28%) after 3 nights. Only the 50 WAKE-24 h mDES scales (one per participant) referring to the day before the recalled dream were included in data analyses.

Twenty-seven participants (54% of the sample) reported a PSQI > 5 and were thus classified as PS [35], while the remaining 23 subjects (46%) made up the GS group. GS and PS were similar in terms of age (GS: 25.26 ± 7.39 vs. PS: 23.96 ± 5.04, t = 0.734, *p* = 0.466, Cohen’s d = 0.21) and gender distribution (GS: 5 M, 18 F vs. PS: 7 M, 20 F, χ^2^_1_ = 0.119, *p* = 0.730), while they significantly differed in PSQI scores (GS: 3.70 ± 0.92 vs. PS: 7.93 ± 1.83, t = −10.496, *p* < 0.001, Cohen’s d = −2.837).

### 3.2. MADRE Scores in Good and Poor Sleepers

The two groups showed similar dream frequency (median = 4, W = 301, *p* = 0.858), intensity of the dream experience (median = 2.5, W = 307.5, *p* = 0.959), attitude towards dreams (median = 2.4, W = 703.5, *p* = 0.770) and correlates of the dream experience (mean = 11, W = 286.5, *p* = 0.647). A significant difference was observed for the frequency of lucid dreams (W = 194.5, *p* = 0.023), with higher frequency in PS (median = 4) compared to GS (median = 3). The frequency of nightmares was nominally higher (W = 224.5, *p* = 0.091) in PS (median = 3) relative to GS (median = 4).

### 3.3. Characterisctics of Dream Emotions

#### 3.3.1. Good Sleepers

In GS, scores at the PA and NA subscales of the DREAM mDES showed a higher intensity of negative emotionality in the dream (PA: 0.80 ± 0.58 vs. NA: 1.40 ± 1.30; t22 = −2.29, *p* = 0.032, Cohens’ d = −0.48).

**Figure 2:**
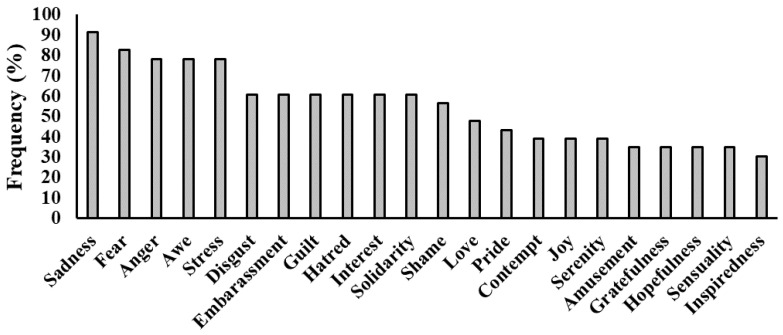
Proportion of Good Sleepers reporting each of the 22 emotions during the dream.

Looking at the specific emotions, all dreams contain at least 8 emotions and all of the 22 emotions are reported at least once. On average, GS reported 12.08 ± 4.79 dream emotions. As displayed in Figure 2, the most frequent emotion is Sadness (reported by 91.3% of the participants), followed by Fear (82.6%) and Anger (78.3%), while the least frequent are Sensuality (30.4%) and Inspiredness (30.43%).

**Figure 3:**
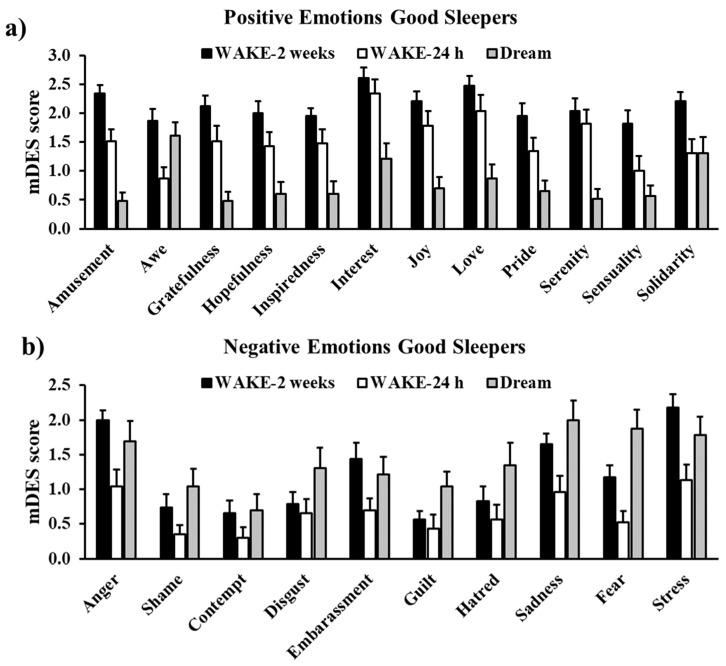
Scores of each emotion in the WAKE-2 weeks, WAKE-24 h, and DREAM mDES in Good Sleepers. Panels (**a**,**b**) display positive and negative emotions, respectively. Error bars represent standard error of the means.

The most intensely experienced emotions during the dream were mostly negative (Figure 3): Sadness (2.00 ± 0.28) was followed by Fear (1.87 ± 1.32), Stress (1.878 ± 1.28), Anger (1.70 ± 1.40), and Awe (1.61 ± 1.12).

#### 3.3.2. Poor Sleepers

Scores at the PA and NA subscales of the DREAM mDES did not differ (PA: 1.10 ± 0.76 vs. NA: 1.13 ± 0.81; t26 = −0.10, *p* = 0.925, Cohens’ d = −0.02), indicating an equal intensity of positive and negative emotionality in the dreams of PS.

**Figure 4:**
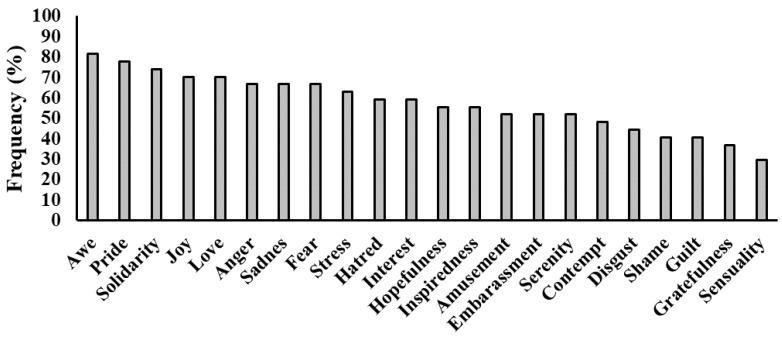
Proportion of Poor Sleepers reporting each of the 22 emotions during the dream.

As for specific emotions, all dreams contain at least 5 emotions and all of the 22 emotions are reported at least once. On average, PS reported 12.63 ± 4.39 dream emotions. As displayed in Figure 4, the most frequent emotion is Awe (reported by 81.5% of the participants), followed by Pride (77.8%) and Solidarity (74.1%), while the least frequent are Gratefulness (37.04%) and Sensuality (29.6%).

**Figure 5:**
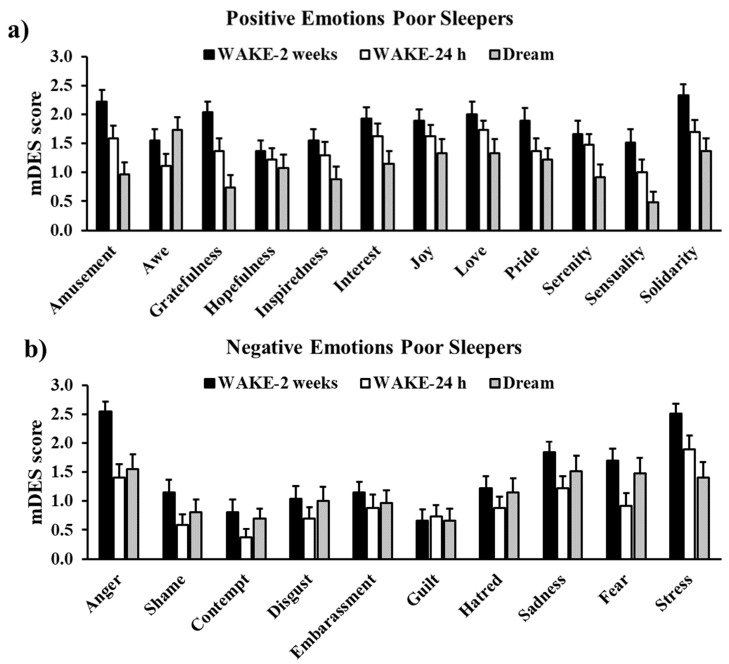
Scores of each emotion in the WAKE-2 weeks, WAKE-24 h, and DREAM mDES in Poor Sleepers. Panels (**a**,**b**) display positive and negative emotions, respectively. Error bars represent standard error of the means.

Although PA and NA scores did not differ, the most intensely experienced emotions during the dream were mostly negative (Figure 5): Awe (1.74 ± 0.22) was followed by Anger (1.55 ± 0.25), Sadness (1.52 ± 0.26), Fear (1.48 ± 0.27), and Stress (1.41 ± 0.27).

### 3.4. Differences between Waking and Dream Emotions in Good and Poor Sleepers

**Figure 6:**
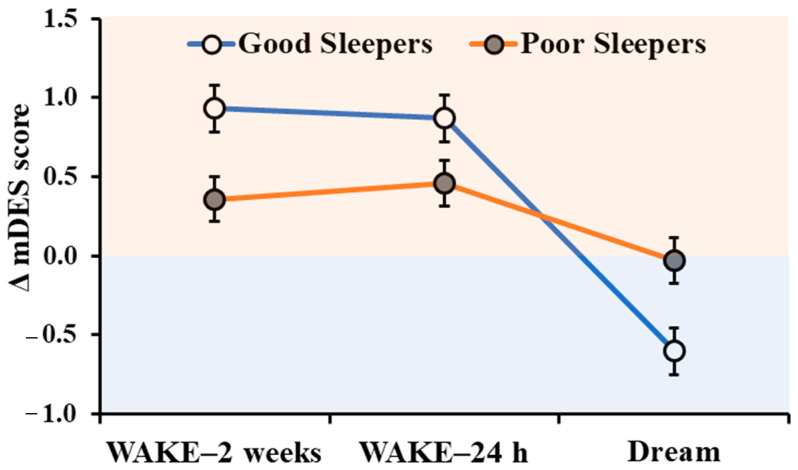
Change in Δ mDES scores (PA minus NA) as a function of condition (WAKE-2 weeks, WAKE-24 h, and DREAM) in Good and Poor Sleepers. The orange area indicates positive affect and the blue area indicates negative affect. Error bars represent standard error of the means.

The ANOVA on Δ mDES scores yielded a significant main effect of condition (F_2,96_ = 15.41, *p* < 0.001, η_p_^2^ = 0.24), with a decrease of delta scores (i.e., more negative emotionality) in the DREAM compared to WAKE-2 weeks and WAKE-24 h (all p_holm_’s < 0.001), and no difference between WAKE-2 weeks and WAKE-24 h (p_holm_ = 0.923). Although we did not find a main effect of Group (F_1,48_ = 0.40, *p* = 0.528, η_p_^2^ < 0.01), we observed a significant Group × Condition interaction (F_2,96_ = 4.72, *p* = 0.011, η_p_^2^ = 0.09, Figure 6): only GS displayed a reduction of delta scores from wakefulness to dream (WAKE-2 weeks vs. DREAM: p_holm_ < 0.001; WAKE-24 h vs. DREAM: p_holm_ < 0.001), while PS did not show any significant change (all p_holm_’s > 0.644). No between-groups differences were observed in any of the three conditions (all p_holm_’s > 0.643, Table 1).

### 3.5. Predictors of Dream Emotional Valence (Δ mDES Scores) in Good and Poor Sleepers

In GS, linear regression analysis showed that neither WAKE-2 weeks nor WAKE-24 h Δ scores were predictive of DREAM Δ scores (F_2,20_ = 0.04, *p* = 0.952, Adj. R2 < 0.01). The same result was observed in PS (F_2,24_ = 0.99, *p* = 0.387, Adj. R2 < 0.01).

## 4. Discussion

This study investigated the relationships between dream emotions and those experienced during the previous days (both the day before the recalled dream and over the two weeks preceding it) in good and poor sleepers. In the frame of theoretical models on the role of dreaming in emotion regulation, postulating a close link between waking and dream emotionality, we aimed to assess the influence of poor sleep quality on this relationship. In fact, though previous literature has already shown the prevalence of negatively toned dreams in populations with disturbed sleep, we believe that affect regulation processes are plausibly better expressed in the interplay between waking and dream emotions rather than in dream emotions alone.

### 4.1. Proportion of Good and Poor Sleepers

Before discussing our main results, it is worth commenting on the high proportion of poor sleepers that emerged in our sample (54%). Considering that most of our participants were university students (mean age: 24.6 ± 6.4 years), this result is in line with those of several wide survey studies assessing the prevalence of poor sleep quality through the PSQI on similar populations and age groups. Indeed, the proportion of poor sleepers was over 40% in Mah et al. [55] and exceeded 60% in Lund et al. [56] and Becker et al. [57].

### 4.2. Results from the MADRE Questionnaire in Good and Poor Sleepers

Data from the MADRE questionnaire show that GS and PS are similar in most dream related variables, including dream frequency, intensity of dreams, attitude towards dreams, and perceived effects of dreams on waking life problem solving and creativity skills. However, PS show a higher frequency both of nightmares and lucid dreams. As for nightmares, this finding is consistent with previous studies showing increased nightmare frequency in poor sleepers [40,41,42,43]. Also, lucid dreaming has sometimes been associated with disrupted sleep [58,59]. Interestingly, nightmares and lucid dreaming have been conceptualized as belonging to a common domain involving unusual cognitions and perceptions in wakefulness and sleep [60], which would be linked to arousal and hypervigilance intruding in the sleep state [58,61] and thus could be viewed as indicators of poor sleep quality [58]. In other words, these hypotheses point to the existence of a close link between the quality of physiological sleep features and that of subjective sleep mentation.

### 4.3. Frequency and Valence of Dream Emotions in Good and Poor Sleepers

GS and PS reported on average a similar number of dream emotions (slightly more than 12), suggesting that the average amount of emotions (12.38) found in our previous study on the whole sample [15] was not affected by sleep quality. The number of emotions in our two samples is slightly higher than that reported in previous literature using the same self-report scale [62], probably because of methodological differences (see [15]).

As for emotional valence, GS displayed higher negative than positive emotionality (scores at the NA subscale) in the dream, whereas, in PS’ dreams, positive and negative emotionality appeared with equal intensity (no difference between PA and NA scores of the DREAM mDES). This finding well accounts for the one emerged in our previous study [15], in which NA scores were slightly higher than those at the PA subscale, but the difference failed to reach significance. The higher negative affect observed in GS is coherent with the finding that specific negative emotions were the most frequent as well as the most intense in this sample; also, it is in line with several previous studies showing a prevalence of negative emotions in dreams (e.g., [63,64,65,66], but see also [67] for a discussion on the differences between self and external ratings of dream emotions). Instead, the evenly distributed emotional tone observed in PS’ dreams apparently contradicts existing literature on populations with sleep impairments [36,37,38,39,40,41,42,43], which points to more negatively toned dreams in these individuals. However, our analysis of specific emotions showed that, although positive emotions were the most frequently reported by PS, their negative emotions were the most intense. In addition, it must be considered that: (a) the instrument we used is quite different from those commonly used in dream research, since it includes a much broader repertoire of emotions and a more balanced number of positive and negative items, thus reducing the risk of underestimating the presence of positive emotionality; (b) results obtained on sleep disordered populations [36,37,38,39] are not fully comparable to those observed in healthy samples reporting poor sleep quality; (c) the higher frequency of nightmares observed in individuals with poor sleep quality (both in our present study and in previous research [40,41,42,43]) does not necessarily imply that their dreams are generally more negatively valenced (in fact, emotionality may be viewed as a “tonic” feature of sleep mentation, while nightmares, or lucid dreams, may be better conceptualized as “phasic” events, although the notion of a continuum between bad dreams and nightmares is sustained by several authors [68,69]).

### 4.4. Relationships between Waking and Dream Emotions in Good and Poor Sleepers

The main finding of our study is the difference observed between GS and PS in the profile of waking and dream emotionality. While GS display a striking inflection of emotional tone from wakefulness to the dream (i.e., affective tone is prevalently positive both during the previous weeks and the previous day and becomes extremely negative in the dream), PS’ emotionality remains stable across conditions. Specifically, in PS, differences between positive and negative emotionality (i.e., delta values) are very close to zero in all three scales.

First of all, this pattern of data suggests that habitual sleep quality significantly affects the interplay of emotional expression across wakefulness and dreaming. This observation is particularly important in light of the numerous discrepancies existing in data on dream features and especially dream emotionality (see [62,67,70]). Controversial results in this field are usually explained through methodological biases as well as biases linked to the retrospective nature of dream descriptions [62,67,70]. Our data prompt us to consider sleep quality as an additional factor affecting dream emotional experience, and thus able to confound results when not controlled for. Therefore, we believe that future dream investigations should include assessments of sleep quality even when addressing nonclinical samples.

At the theoretical level, the differences observed between GS and PS appear to reflect a different functionality of sleep–wake emotion regulation processes, in line with the recent models on dream-related affect regulation [1,3,11,12,13,14]. In other words, the lack of oscillations in prevalent emotional valence of PS (expressed by their flattened curve of Δ mDES scores across daytime and sleep) may depend on a relative impairment of their emotion regulation processes, whose effectiveness would instead be expressed by the opposite emotional tone in wakefulness and dreams of GS. Specifically, GS display a prevalence of positive affect during daytime and negative affect during the dream. As suggested in Conte et al. [15], the negative emotions experienced more frequently or intensely in the general period in which the dream occurs would be those in need of regulation during sleep, whereas positive emotions, requiring less modulation, would be underrepresented in the dream. Also, the predominant positive affect observed during wakefulness in GS would at least partly depend on effective sleep-related modulation that occurred in previous dreams. As for PS, they showed lower positive emotionality than GS both during the two weeks and the day preceding the dream (although the differences between groups did not reach significance). This observation is in line with past literature showing lower well-being and positive affect in poor sleepers [30,31,32,71], which also is plausibly linked to less effective sleep-related affect modulation. Moreover, as suggested above, the fact that negative affect does not prevail in PS’ dreams could indicate poor functioning of sleep-related emotion regulation.

A complementary explanation may also be proposed, referring to the recent hypothesis of a dream rebound of thoughts suppressed during wakefulness [72,73,74], which, in turn, can be traced back to Freud’s idea [75] that dreams reflect the return of mental contents inhibited during the waking hours. This kind of mechanism was plausibly active in GS, whose negative emotions, excluded from waking consciousness in favor of positive ones, may have rebounded in the dream. The process of negative affect suppression could instead have been ineffective in PS, possibly due to the fact that disrupted sleep is linked to deficits in higher cognitive functions including inhibition (e.g., [19,76,77]). In line with Malinowski et al. [78], who showed that successful suppression of thoughts and their rebound in the dream benefit the emotional response to pleasant and unpleasant thoughts, it may be hypothesized that, in good sleepers, the dream rebound of negative emotions reflects their effective processing in sleep, irrespective of the specific episodic memories (thoughts, events, etc.) that generated them.

In sum, our findings are probably the result of two parallel mechanisms: a general day-night emotion regulation process (with prevalent negative emotions of daytime being processed in sleep and thus reappearing in dreaming) and the specific suppression (either deliberate or automatic) of certain negative emotions during wakefulness with consequent rebound in the dream for regulation purposes.

It must be acknowledged here that our regression analysis on waking and dream delta scores did not yield significant results. In fact, emotional tone of the previous day and previous two weeks did not predict that of the dream in either group of participants. This result is consistent with three other studies which found few [79], small [80], or no correlations [81] between corresponding dream and previous daytime emotions. The absence, to date, of data on direct relationships between waking and dream affect does not lend support to our main hypothesis, i.e., the interpretation of our data in the frame of theories on dream-related emotion regulation [1,3,11,12,13,14]. However, clearer associations between waking and dream affect could exist across different time spans and in different directions than those investigated here and in the abovementioned studies [79,80,81]. In fact, as pointed out in the introduction, each dream could process emotions experienced the day before, a few days before (in analogy with literature on the “dream lag” and “day-residue” effect [44,45]), or during wider daytime spans (e.g., the last few weeks, the general “time period”, etc.). Also, as predicted by the “simulation models” [13,14], dream emotionality could reveal stronger associations with future rather than past waking affect, a possibility to be investigated in forthcoming studies.

Furthermore, it may be speculated that poor sleepers rate their dreams as less negatively toned compared to good sleepers also because of a different general perception of the dreaming experience. In other words, they could retrospectively evaluate their dream experience as more positive than it actually was since the simple fact of having dreamed, per se, represents for them a sign of having slept well (good sleepers would obviously have no such bias). This interesting possibility could be usefully investigated in future research.

Finally, our findings allow us to extend the discussion of our previous work on the same sample [15], by underlining the influence exerted by poor habitual sleep quality on waking and dream emotional expression. In fact, here we observed that the opposite prevalent emotional tone of wakefulness and dreams, emerged in the previous study, well describes GS’ profile, while PS display an equal amount of positive and negative affect in both states. The hypotheses made on these findings are coherent with the main interpretations discussed in our previous work. However, the current data allow us to exclude a couple of alternative explanations advanced on those data [15]. Specifically, we proposed that participants may have undergone some sort of social desirability effect in compiling the scales (see, e.g., [82]); in other words, they would have more easily identified positive emotions (coherent with a positive image of the self) in wakefulness and negative emotions in the dream (which is experienced as “involuntary”). Similarly, we acknowledged a possible recall bias linked to the time frame of events to which the emotions refer. In the DREAM mDES, the participant is focusing on a much shorter time frame compared to those of the daytime scales (2 weeks and 24 h). Among this limited pool of memories, the negative ones could appear more salient and thus be more easily recognized (according to the widely held tenet in psychology that “bad is stronger than good” [83]). While these two hypotheses may have applied to our GS group, we see no reason why PS would not have equally undergone these types of biases: thus, the different emotional profile emerged in the latter group induces us to rule out these possibilities.

### 4.5. Limitations

Our results should be considered in light of some limitations to be overcome in future research. The main limitation is the use of a self-report measure of sleep quality rather than standard polysomnography for the identification of good and poor sleepers. However, it must be noted that groups of good and poor sleepers classified through the PSQI have been shown to significantly differ in polysomnographic sleep measures in several previous studies [35,84,85].

Furthermore, according to some authors [67,86], self-ratings of dream emotions based on emotion rating scales may be biased by demand characteristics of the rating task (i.e., individuals may be primed by answer options) or phenomena such as the positivity offset (i.e., the tendency to experience mildly positive mood most of the time); still, several authors argue that self-ratings more validly represent dream emotional experiences [65,87].

## 5. Conclusions

In conclusion, to the best of our knowledge, this is the first study to investigate differences between good and poor sleepers in the profile of emotionality across wakefulness and dreaming. Overall, our findings show that good sleepers experience a notable change in emotionality between wakefulness and dreaming, with a prevalence of positive affect during daytime and predominant negative affect during dreaming, whereas poor sleepers are characterized by equal intensity of positive and negative emotionality in both states. In the frame of recent theoretical models postulating a role of dreaming in affect regulation, the lack of changes in prevalent emotional valence across states observed in the latter group may be interpreted as reflecting ineffective sleep-related emotional processing. Furthermore, regardless of the theoretical framework, our results highlight that sleep quality is associated with notable differences in the expression of waking and dream emotions which should not be neglected in future dream research. Therefore, our findings definitely encourage researchers to include sleep quality assessments in dream studies (both on clinical and nonclinical samples) and prompt future investigations on sleep-impaired populations as a privileged object of study in the field of research on dreaming and emotion regulation processes.

## Figures and Tables

**Table 1:** Mean and standard error of Δ scores in the two groups across conditions.

	Good Sleepers	Poor Sleepers	*p*(holm)
Δ Score			
WAKE-2 weeks	0.932 ± 0.229	0.358 ± 0.219	0.644
WAKE-24 h	0.869 ± 0.229	0.461 ± 0.219	0.694
DREAM	−0.605 ± 0.229	−0.03 ± 0.219	0.644

## Data Availability

The data presented in this study are available on request from the corresponding author. The data are not publicly available due to privacy reasons.
